# Identification of Genomic Regions Regulating Pax6 Expression in Embryonic Forebrain Using YAC Reporter Transgenic Mouse Lines

**DOI:** 10.1371/journal.pone.0080208

**Published:** 2013-11-06

**Authors:** Da Mi, Yu-Ting Huang, Dirk A. Kleinjan, John O. Mason, David J. Price

**Affiliations:** 1 Centre for Integrative Physiology, School of Biomedical Sciences, University of Edinburgh, Edinburgh, United Kingdom; 2 Medical Research Council Human Genetics Unit, Institute of Genetics and Molecular Medicine, University of Edinburgh, Edinburgh, United Kingdom; CNRS UMR7275, France

## Abstract

The transcription factor Pax6 is a crucial regulator of eye and central nervous system development. Both the spatiotemporal patterns and the precise levels of *Pax6* expression are subject to tight control, mediated by an extensive set of cis-regulatory elements. Previous studies have shown that a YAC reporter transgene containing 420Kb of genomic DNA spanning the human *PAX6* locus drives expression of a tau-tagged GFP reporter in mice in a pattern that closely resembles that of endogenous *Pax6*. Here we have closely compared the pattern of tau-GFP reporter expression at the cellular level in the forebrains and eyes of transgenic mice carrying either complete or truncated versions of the YAC reporter transgene with endogenous Pax6 expression and found several areas where expression of tau-GFP and Pax6 diverge. Some discrepancies are due to differences between the intracellular localization or perdurance of tau-GFP and Pax6 proteins, while others are likely to be a consequence of transcriptional differences. We show that cis-regulatory elements that lie outside the 420kb fragment of *PAX6* are required for correct expression around the pallial-subpallial boundary, in the amygdala and the prethalamus. Further, we found that the YAC reporter transgene effectively labels cells that contribute to the lateral cortical stream, including cells that arise from the pallium and subpallium, and therefore represents a useful tool for studying lateral cortical stream migration.

## Introduction

*Pax6* encodes a highly evolutionarily conserved transcription factor with two DNA-binding domains - a paired domain and a paired-type homeodomain [1,2]. *Small eye* (*Sey*) mutant mice heterozygous for a loss-of-function allele of *Pax6* have defective eye development while *Pax6* ^*-/-*^ homozygotes die around the time of birth with multiple defects of the eyes and brain. Humans who are heterozygous for mutations in *PAX6* suffer from congenital aniridia and brain defects while homozygous mutations in PAX6 are incompatible with life [3-9]. *Pax6* is expressed in the eye, pancreas and several regions of the nervous system including the forebrain [7,10-14]. In the forebrain, Pax6 is expressed in a complex and dynamic pattern, including a clear gradient of expression across the developing cerebral cortex. Graded Pax6 levels across the embryonic neocortex are essential for the correct specification of cortical areas and for maintaining their progenitor cell proliferation rates [15-20]. Interestingly, both downregulation and overexpression of Pax6 in mice cause defects of the eye and brain [15-17,20,21]. Thus, tight control of *Pax6*’s complex spatiotemporal expression pattern is essential for normal development. 

**Figure 1:**
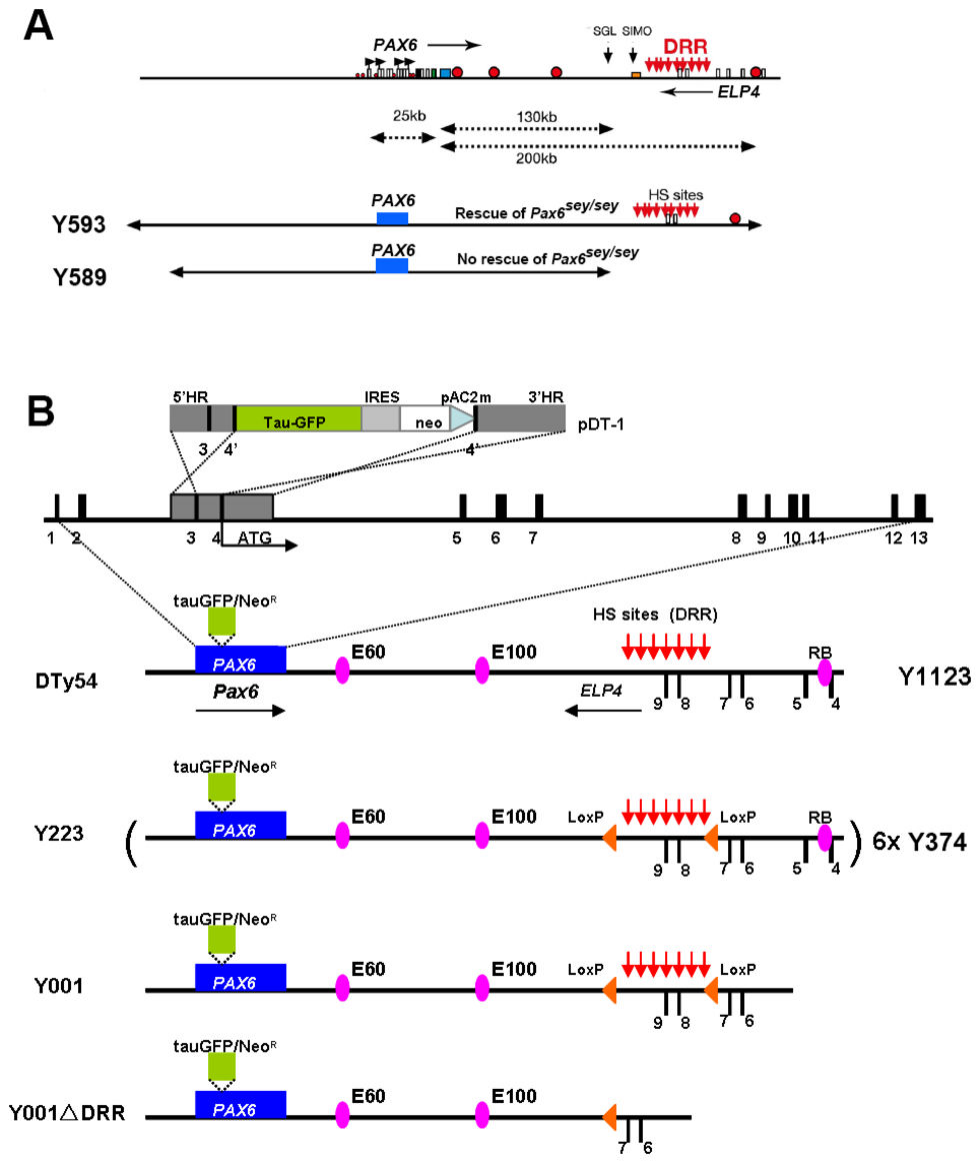
Summary of YAC transgenic mouse lines. (A) Schematic map of human chromosome 11p13 showing the locations of the *PAX6* and *ELP4* loci, which are in an antisense orientation relative to each other. The locations of PAX6 promoters (P0, P1, Pa and P4) are indicated by black arrows. *PAX6* and *ELP4* exons are shown as open rectangles. The breakpoints of the two distal-most aniridia-associated rearrangements are indicated by “SGL” and “SIMO”. Red circles indicate some known *PAX6* regulatory elements. The location of the downstream regulatory region (DRR) is shown by red arrows. The schematic maps of YACs Y593 (420 kb) and Y589 (310 kb) are indicated. In transgenic mice, Y593, but not Y589, rescues the mouse *Sey* phenotype and homozygous *Sey* lethality. (B) Schematic map of YAC transgenes in different YAC transgenic mouse lines. Construct pDT-1 contained sequences expressing tau-GFP and conferring neomycin (neo) resistance, separated by an internal ribosomal entry site (IRES), terminated with polyadenylation and C2MAZ (pAC2m) sequences and flanked by 5' and 3' homology regions (5'HR and 3'HR). Homologous recombination resulted in the introduction of this construct at the translation initiation site (ATG) in exon 4 of the human *PAX6* locus, to produce the YAC Y1123.. The *PAX6* transcription unit is indicated by a blue box into which a tau-GFP-IRES-Neomycin cassette, indicated by green box, was inserted. The known hypersensitive sites (HS sites) within the DRR region are shown by red arrows. The position of *LoxP* sites are indicated by orange triangles. The highly conserved *cis*-regulatory elements E60, E100 and RB are indicated by purple ellipses. Line DTy54 contains a single-copy of YAC 1123. Line Y223 carries 6 copies of YAC 374 in which a pair of *LoxP* sites flank a 35 kb genomic region within the DRR. Line Y001 contains a single copy of the truncated YAC 374 lacking the distal-most 20 kb sequences including the known RB enhancer. Line Y001ΔDRR is directly derived from Y001 line by Cre-mediated deletion of the 35 kb DRR region. All YACs have the same sequences at the 5’ end.

A number of previous studies have examined mechanisms involved in the transcriptional control of *Pax6*. Several short-range cis-regulatory elements have been identified 5’ and 3’ of the *Pax6* transcript unit and in its introns and have been characterized using reporter assays in transgenic mice [9,22-29]. Notably, some of these regulatory elements exhibit overlapping tissue specificity, particularly in the eye and diencephalon, suggesting that they have cooperative functions. Although these short-range regulatory elements account for much of the endogenous *Pax6* expression pattern in mice, genetic analyses from humans and mice revealed that they are insufficient to drive the full pattern of *Pax6* expression, particularly in the eye and brain [27,30,31]. Evidence for the presence of distant regulatory elements downstream of human *PAX6* came from genetic analyses of patients with congenital aniridia, some of whom have chromosomal rearrangements whose breakpoints are far downstream of the *PAX6* transcriptional unit [32-36], suggesting that the rearrangement has separated distal regulatory elements from the body of the *PAX6* gene, leading to defective *PAX6* expression. The most distant breakpoint of these aniridia-related chromosomal rearrangements, designated “SIMO” (Figure 1A), is located 124 kb downstream from the *PAX6* polyadenylation site [32,34]. The importance of this distant region was reinforced by the finding that a 420 kb yeast artificial chromosome (YAC) Y593 (Figure 1A) comprising the human *PAX6* coding sequence and 200 kb flanking genomic regions on either side, stretching beyond the SIMO breakpoint, rescues the eye phenotype of *small eye* (*Sey*) mice, whereas a shorter YAC transgene Y589 (Figure 1A) containing 110 kb less of Y593’s DNA sequence (30 kb at the 5’ end and 80 kb at the 3’ end ) does not [34]. Further detailed analyses through genomic sequence comparison and DNaseI hypersensitivity mapping suggested the presence of regulatory elements within the *PAX6* downstream regulatory region (DRR) extending over 75 kb mainly 3’ to the SIMO breakpoint (Figure 1A) [21,31,34]. These elements are located within introns of the adjacent, ubiquitously expressed gene *ELP4* and are thought to be crucial for the induction of *PAX6* expression in eye and diencephalon [27,28,30,31,37].

Previous work aimed at gaining a better insight into the functions of distant regulatory elements in governing *Pax6* expression generated a series of novel YAC transgenic reporter mice, carrying either full length 420 kb YAC 593 or truncated YAC transgenes in which a tau-GFP reporter cassette replaces the first coding *PAX6* exon, preventing the production of functional full-length PAX6 from the transgenes. In these transgenic lines, the GFP reporter cassette is inserted at the *PAX6* translational start site in exon 4 (Figure 1B). GFP expression is, therefore, under the control of those *PAX6* regulatory elements contained within the YAC transgene and is independent of the endogenous mouse *Pax6* locus [30,31,38]. Previous analyses of these lines did not examine the detailed expression patterns of the transgenes in sub-regions of the eye and brain. Here, we carried out such an analysis since relatively subtle deviations from normal patterns of endogenous *Pax6* expression are likely to provide further useful insights into *Pax6* regulation and to be important for those using these transgenic alleles as tools to mark specific populations of cells. 

## Materials and Methods

### Ethics statement

The licence authorising this work was approved by the University of Edinburgh's Ethical Review Committee on 22nd September 2008 (application number PL35-08) and by the Home Office on 6th November 2008. Animal husbandry was in accordance with the UK Animals (Scientific Procedures) Act 1986 regulations. To minimise animal suffering, pregnant dams were culled by cervical dislocation under terminal anaesthesia according to the Code of Practice for Humane Killing of Animals under Schedule 1 to the Animals (Scientific Procedures) Act 1986 issued by the Home Office.

### Mice

Mice were bred in accordance with the UK Animals (Scientific Procedures) Act 1986 regulations. YAC transgenic mouse lines DTy54, Y223, Y001 and Y001ΔDRR were hemizygous for the tau-GFP reporter YAC transgene and were maintained on a CD1 background. Transgenic animals were identified by PCR for the reporter transgene as described previously [38] and embryos were genotyped by detecting GFP fluorescence. 

### Immunofluorescence

Following deparafinization in xylene, sections were rehydrated through a descending ethanol series and washed in PBS. Antigen retrieval was carried out by microwaving sections in 0.01M sodium citrate (pH6.0) for 20 minutes. Sections were washed with PBS-0.01% Triton X-100 and then incubated for 30 minutes in blocking solution containing 20% goat or donkey serum, then incubated overnight at 4°C with primary antibodies diluted in blocking solution. Primary antibodies used in this study were rabbit anti-GFP (Abcam 1:100), mouse anti-Pax6 (DSHB 1:100), rabbit anti-Tbr1 (Abcam 1:100), rabbit anti-Tbr2 (Abcam 1:100), rabbit anti-Gsh2 (gift from Kenneth Campbell 1:1500). Following incubation with primary antibodies, sections were washed in PBS-0.01% Triton X-100 and incubated with secondary antibodies for 1 hour at room temperature. For fluorescent staining, secondary antibodies used were donkey anti-mouse Alexa Fluor 488 or 568 as well as donkey anti-rabbit Alexa Fluor 488 or 568. Nuclei were counterstained with TOPRO-3 (Molecular Probes, 1:1000). Fluorescent images were captured using a Leica NTS confocal microscope.

### In situ hybridization

In situ hybridization was performed using the standard protocol [39]. Sense and antisense digoxigenin-labeled RNA probes were generated from a BamHI digest of the pCR-Blunt II-TOPO *Pax6* cDNA clone and a *Kpn1* digest of the pBluescript II KS+ *GFP* cDNA clone using a DIG RNA labeling kit (Roche). Sections were dewaxed and rehydrated through xylene and graded alcohols, washed in PBS and digested in 20µg/ml Proteinase K in PBS at 37°C for 5 minutes. Sections were then washed first in 0.2% Glycine/PBS and then in PBS, and post-fixed in 4% PFA/0.2% glutaraldehyde for 20 minutes. Sections were washed again in PBS before incubation with prehyb solution for two hours and were then hybridized overnight with 1ng/µl probe in prehyb solution. Following hybridization sections were rinsed in 2xSSC, pH4.5 and then washed at 5°C below the hybridization temperature in 50% Formamide/2xSSC. Sections were then washed in PBS/Tween^0.1%^, incubated for 1 hour at room temperature in Boehringer blocking solution containing 10% sheep serum followed by incubation at 37°C with anti-DIG for 2 hours. Sections were washed overnight in PBS/Tween^0.1%^, and finally washed in NTM and stained with NBT/BCIP.

## Results

### Detailed comparison of tau-GFP reporter and endogenous Pax6 expression in the forebrain of DTy54 transgenic mice

DTy54 transgenic mice carry a single copy of YAC 1123, which is a full-length version of YAC 593 modified so that it expresses tau-tagged GFP instead of PAX6 (summarized in the upper part of Figure 1B) [38]. To compare expression of the tau-GFP reporter and endogenous Pax6, coronal sections along the rostral–caudal axis were double-immunolabeled for tau-GFP and Pax6 protein. Whereas staining for Pax6 is nuclear, tau-GFP binds to the microtubule component of the cytoskeleton and labels the cytoplasm [38,40]. 

**Figure 2:**
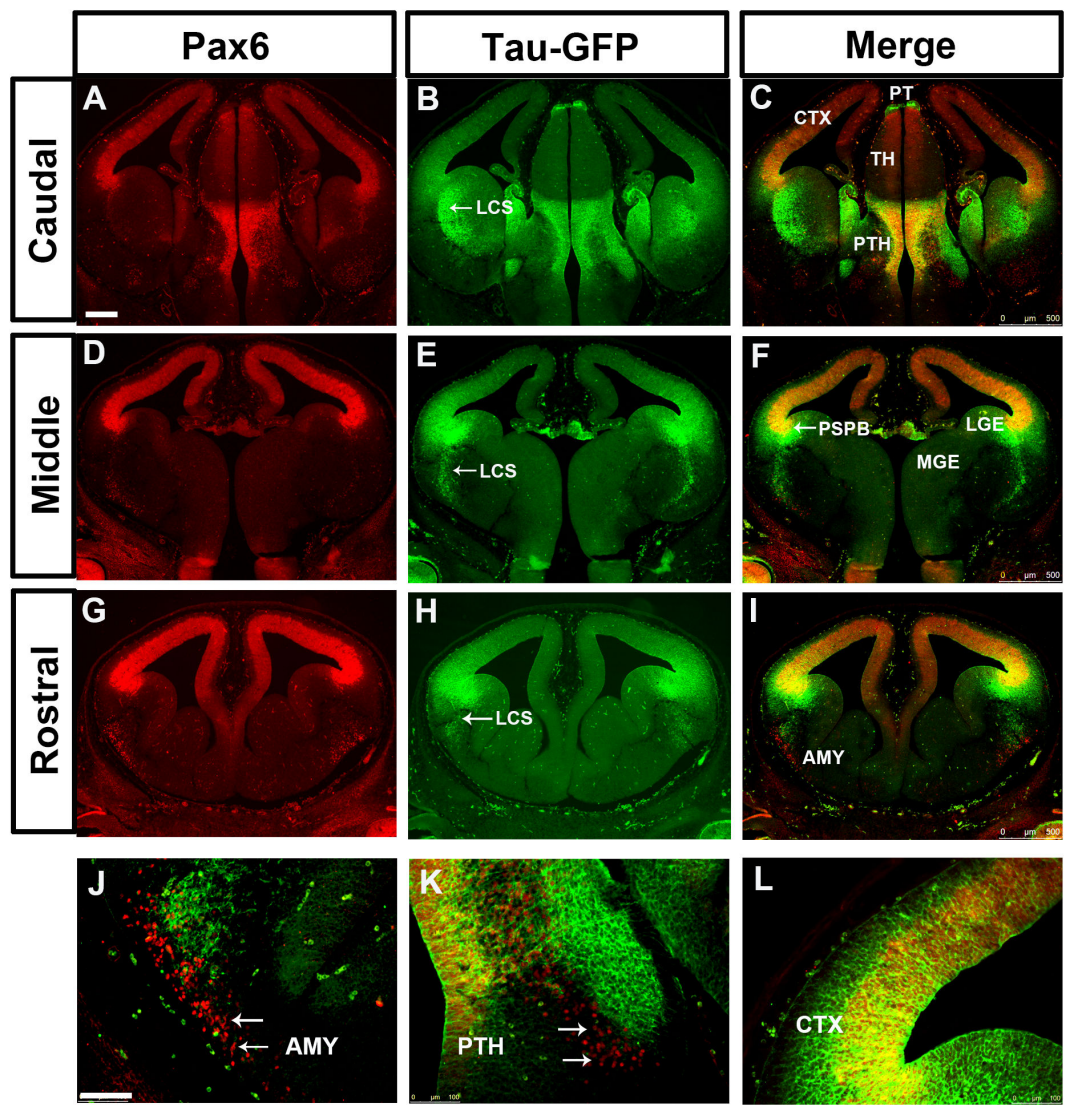
Expression of Pax6 and tau-GFP in DTy54 embryonic forebrain at E12.5. Double immunofluorescence for Pax6 (red) and GFP (green) on coronal sections taken along the rostrocaudal axis. (A, D, G) In the telencephalon, Pax6 is expressed in the ventricular zone of the cortex (CTX), in the VZ of LGE with very low levels, in a stream of cells migrating towards the basal ventral telencephalon, and in the amygdaloid area (AMY). In the diencephalon, Pax6 is broadly expressed in the pretectum (PT) and prethalamus (PTH) with high expression levels, and in the midline of the thalamus (TH) with relatively low levels. (B, E, H) tau-GFP is expressed in all Pax6 expressing regions. (C, F, I) Direct comparison of tau-GFP and Pax6 expression revealed that tau-GFP expression replicates the known Pax6 pattern with some exceptions. (J) Pax6 single expressing cells are present within the amygdaloid region (arrows). (K) A band of Pax6 only expressing cells is located adjacent to the tau-GFP labeled cellular processes in the prethalamus (arrows). (L) tau-GFP labeled radial glial processes extend into the mantle region of the cortex. LCS, lateral cortical stream; PSPB, pallial subpallial boundary; LGE, lateral ganglionic eminence; MGE, medial ganglionic eminence. n=10 DTy54 embryos. Scale bars: A-I, 200µm; J-L, 100 µm.

In DTy54 embryos, tau-GFP was seen in regions of the telencephalon known to express Pax6. The expression level of tau-GFP in the cerebral cortex was graded from high laterally to low medially at all rostral-caudal levels and was generally higher rostrally (Figure 2B,E,H), in agreement with the gradient of Pax6 expression (Figure 2A,D,G). However, the coincidence between tau-GFP and Pax6 expression broke down in the subpallium. Whereas there was a step down in the level of Pax6 expression from cortex to LGE at the pallial-subpallial border (PSPB), high levels of tau-GFP extended across the PSPB into the LGE (Figure 2A-I). Pax6 and tau-GFP were expressed in cells migrating ventrally away from the PSPB region along the putative lateral cortical stream (LCS) towards the developing basolateral telencephalon, but the expression domain of tau-GFP in this region was broader than that of Pax6, particularly caudally (Figure 2A,B,D,E,G,H). The expression level of tau-GFP relative to its levels in other tau-GFP-expressing regions was higher than that of Pax6 relative to its levels in other Pax6-expressing regions. We also observed a discrepancy between tau-GFP and Pax6 expression in the amygdaloid area, where a population of Pax6 expressing cells close to the pial surface did not express tau-GFP (Figure 2J).

In the diencephalon, tau-GFP expression mirrored quite closely that of Pax6, with high levels of both in the pretectum, low levels of both in the thalamus and high levels of both in the prethalamus (Figure 2A-C). In the prethalamus, Pax6+ and tau-GFP+ cells were located through the depth of the neuroepithelium from the ventricular surface to the pial surface, with a large amount of tau-GFP on the pial side (Figure 2K). In addition, as in the amygdaloid area, we observed a small patch of ventrally-located Pax6+ cells that were negative for tau-GFP (Figure 2K)

In general, a lack of coincidence between tau-GFP and Pax6 proteins might stem from the differences in their subcellular localization. Pax6+ progenitors in the anterior neural tube have radial processes that span the overlying neuroepithelium. These processes would accumulate tau-GFP but not Pax6 protein. Another factor might be a longer perdurance of tau-GFP protein than Pax6 protein in postmitotic cells that have migrated into the mantle zone from the underlying proliferative zone. These factors could account for tau-GFP staining overlying the Pax6+ progenitors in the cortex and diencephalon (Figure 2K,L). However, neither is likely to explain the unusually high levels of tau-GFP in a broad region of the ventricular zone of the LGE and in postmitotic cells in and around the LCS, given the very low levels of Pax6 protein in the ventricular zone of the LGE and the relative sparseness of Pax6+ cells in the LCS (Figure 2A-I). 

### Comparison of tau-GFP and Pax6 mRNA expression suggests that some regulatory elements are missing from YAC1123

**Figure 3:**
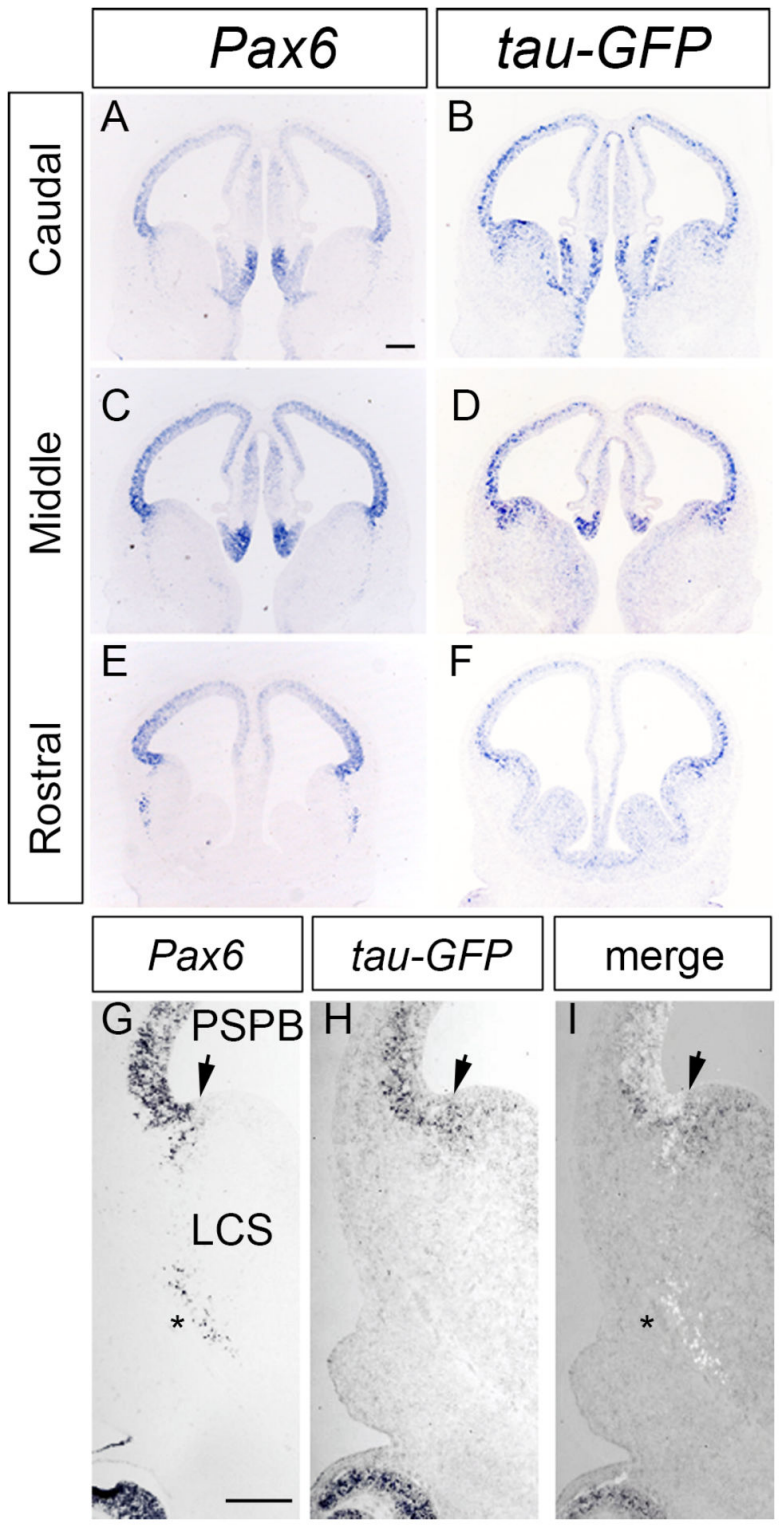
*In* *situ* hybridization for *Pax6* and *Tau-GFP* on coronal sections of E12.5 DTy54 embryos. Adjacent 10μm coronal sections were hybridized with a probe for *Pax6* (A,C,E,G) or *tau-GFP* (B,D,F,H). (A-H) *Pax6* and *tau-GFP* expression throughout caudal to rostral levels of the forebrain. (G-I) Direct comparison of *Pax6* and *tau-GFP* transcripts in the ventral telencephalon. Image in G is colour-inverted and superimposed on that in H to produce the merged image in I. Arrow indicates the PSPB. Strong *tau-GFP* expression was expanded into the subpallial side of the PSPB, while *Pax6* expression levels dropped sharply at the PSPB. In addition, only Pax6 mRNA but not *tau-GFP* mRNA was expressed in the postmitotic cells in the basolateral telencephalon (asterisk in G). LCS, lateral cortical stream. n=8 DTy54 embryos. Scale bars: 200µm.

As described above, the very high levels of tau-GFP in the subpallium (Figure 2B,E,H) are associated with ventricular zone progenitors, their radial process and the postmitotic derivatives of those progenitors, which express either very low or undetectable levels of Pax6. Discrepancies between the patterns of tau-GFP and Pax6 protein expression in the subpallium of DTy54 embryos might arise as a result of differences in transcription from the transgene and the endogenous *Pax6* locus. To address this possibility, we carried out in situ hybridizations for *tau-GFP* and *Pax6* in adjacent sections of E12.5 DTy54 forebrain at all rostral–caudal levels. In the cortex, both transcripts were expressed in the ventricular zone with levels that were graded from high laterally to low medially (Figure 3A-F). But while *Pax6* mRNA levels dropped sharply at the PSPB, levels of *tau-GFP* mRNA did not (Figure 3 G-I), mirroring the discrepancy between the Pax6 and tau-GFP protein expression patterns in this region. In addition, whereas *Pax6* mRNA was expressed in postmitotic cells in the basolateral telencephalon (Figure 3G,I), as was Pax6 protein (Figure 2J), *tau-GFP* mRNA was undetectable in these cells (Figure 3H,I), as was tau-GFP protein (Figure 2J). These results indicate that the discrepancy between the Pax6 and tau-GFP protein expression patterns in the subpallium can be accounted for by difference in transcription between the YAC 1123 transgene and the endogenous *Pax6* locus in this region. 

This analysis also showed that *tau-GFP* mRNA was not expressed by cells in the LCS (Figure 3H), unlike tau-GFP protein expression which was present along the whole LCS (Figure 2 B,E,H). The presence of tau-GFP protein along the LCS is likely explained by its being in the radial processes of progenitor cells around the PSPB (which are known to form a palisade at this boundary that defines the route of the LCS [41,42]) and perdurance of tau-GFP protein in migrating derivatives of these progenitors. 

### Analysis of tau-GFP in the LCS in DTy54 transgenic mice

**Figure 4:**
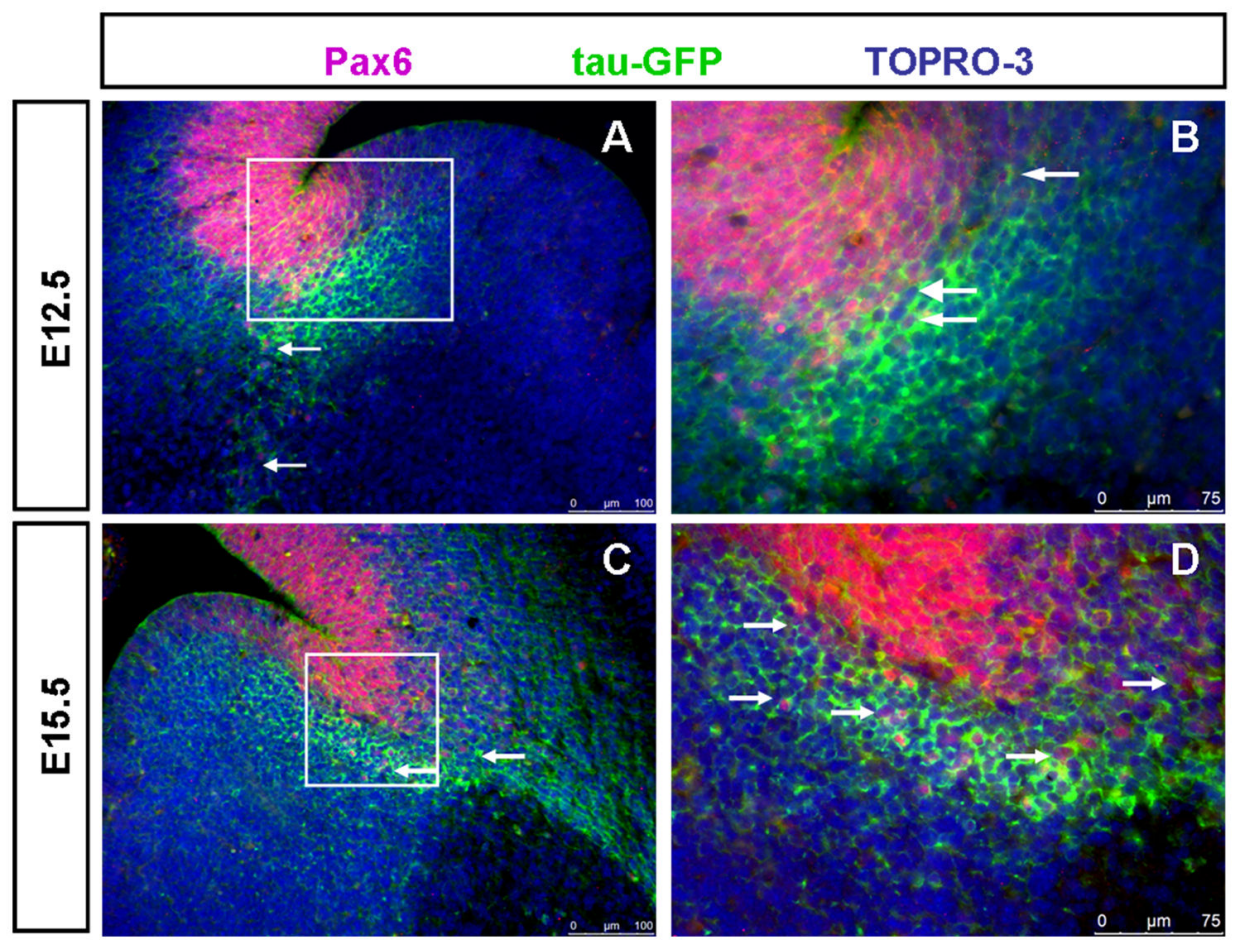
Pax6 is expressed in a subpopulation of tau-GFP heavily labeled LCS in DTy54 transgenic mouse line at both E12.5 and E15.5. (A) Pax6 is expressed in the tau-GFP labeled LCS cells at E12.5 (arrows). (B) Higher magnification of boxed area in A revealed that at the ventricular zone of the PSPB, tau-GFP heavily expressing cells are located adjacent to the Pax6 positive cell populations, along with a small subset of cells expressing both tau-GFP and Pax6 (arrows). (C) Pax6 is expressed in the tau-GFP labeled LCS cells at E15.5 (arrows). (D) Higher magnification of boxed area in C further revealed that a subset of tau-GFP labeled LCS cells also express Pax6 (arrows). n=10 DTy54 embryos.

**Figure 5:**
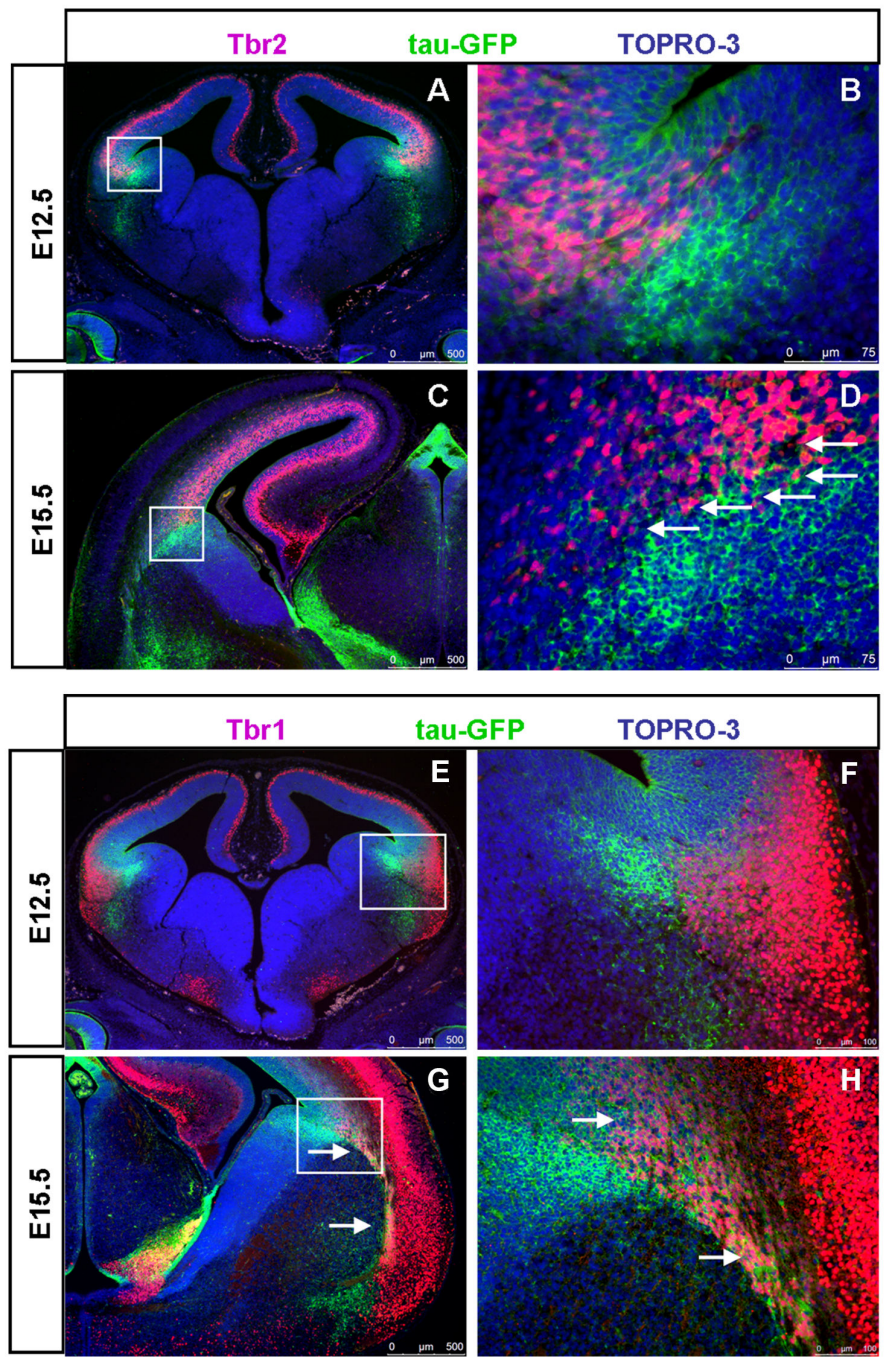
Some tau-GFP labelled LCS cells co-express Tbr2 and Tbr1 in DTy54 transgenic mice. (A) Tbr2 and tau-GFP expression at E12.5. (B) Higher magnification of boxed area in A revealing that Tbr2 positive cells are juxtaposed to the most strongly tau-GFP labeled cells. (C) Tbr2 and tau-GFP expression at E15.5. (D) Higher magnification of boxed area in C further revealed that a subset of tau-GFP labeled LCS cells also expressed Tbr2 (arrows). (E) Tbr1 is not expressed in the tau-GFP labeled LCS cells at E12.5. (F) Higher magnification of boxed area in E revealed that Tbr1 positive cells are distinct to the tau-GFP strongly labeled LCS cells. (G) A subset of tau-GFP labeled LCS cells co-expressed Tbr1 at E15.5 (arrows). (H) Higher magnification of boxed area in G further revealed that a subset of tau-GFP labeled LCS cells, which seem to arise from the mantle region of the pallium, co-expresses Tbr1 (arrows). n=10 DTy54 embryos.

**Figure 6:**
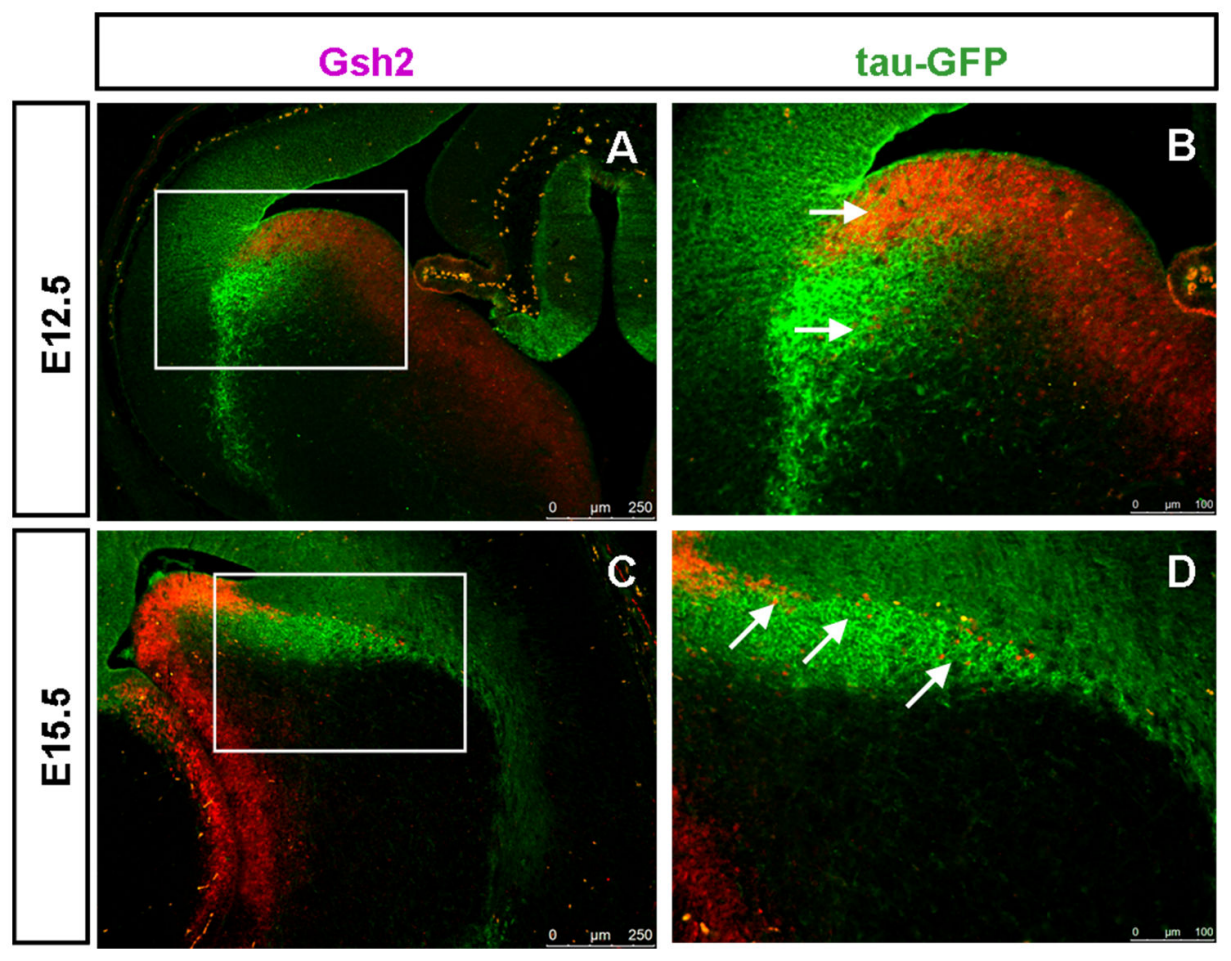
Gsh2 is expressed in a subpopulation of tau-GFP heavily labeled LCS cells in DTy54 transgenic mouse line at both E12.5 and E15.5. (A) The tau-GFP heavily labeled cell populations overlapped the Gsh2 positive domain in the VZ of the subpallium at E12.5. (B) Higher magnification of boxed area in A revealed that a subset of tau-GFP labeled LCS cells co-expressed Gsh2 (e.g. arrows). (C) A stream of Gsh2 expressing cells are present within the tau-GFP labeled LCS at E15.5. (D) Higher magnification of boxed area in C further revealed that a subset of tau-GFP labeled LCS cells co-expresses Gsh2 (e.g. arrows). n=10 DTy54 embryos.

Expression of tau-GFP protein in the LCS of DTy54 embryos was correlated with the expression of other LCS markers using double-immunostaining. Many Pax6/ tau-GFP co-expressing cells were observed on the pallial side of the PSPB at E12.5 and E15.5 (Figure 4). Very few tau-GFP+ cells on the subpallial side of the PSPB expressed Pax6 at E12.5 (Figure 4 A,B). Slightly more of them expressed Pax6 at E15.5, but most did not (Figure 4 C,D). The pallial marker Tbr2 is expressed in basal progenitor cells located in the pallial subventricular zone [43-45] whereas Tbr1, another pallial marker, is expressed in post-mitotic neurons in the mantle layers and migrating along the LCS to contribute excitatory neurons to the amygdala [46-48]. At E12.5 and E15.5, most of the strongly labelled tau-GFP+ cells that lay immediately beneath the ventricular zone of the LGE expressed neither Tbr2 nor Tbr1 (Figure 5). By E15.5, the LCS comprised a mixture of cells that expressed both tau-GFP and Tbr1+ (presumably pallial-derived) or tau-GFP only (presumably subpallial-derived) (Figure 5H). Gsh2 is a subpallial marker expressed by progenitors of the LGE and neurons migrating from this region along the LCS to become excitatory neurons in the amygdala [41,49-51]. At E12.5, the heavily labeled tau-GFP+ subpallial cells lay beneath the Gsh2+ domain in the ventricular zone of the subpallium (Figure 6 A,B). At E15.5, a stream of Gsh2+ cells was present within the tau-GFP labeled LCS (Figure 6 C,D). These Gsh2+ cells ran through the centre of the LCS, dividing it into a ventral part expressing high levels of tau-GFP and a dorsal part expressing lower levels of tau-GFP (Figure 6D). 

These results indicate that the tau-GFP labeled LCS comprises a mixture of cell types, some derived from progenitors on the subpallial side of the PSPB and others from progenitors on the pallial side, subsets of which express Pax6, Tbr2, Tbr1 or Gsh2. Tau-GFP expression in DTy54 mice is a good marker of cells entering and migrating through the LCS. The particularly strong expression of tau-GFP along the LCS on the ventral side of the PSPB is likely to be due to a combination of perdurance of tau-GFP protein and ectopic transcription of the reporter YAC. Such ectopic transcription of the transgene could be due to the absence of one or more transcriptional repressive element(s) from the YAC, or an effect of the transgene integration site. 

### Analysis of tau-GFP reporter expression in the forebrain of other PAX6 YAC reporter transgenics

**Figure 7:**
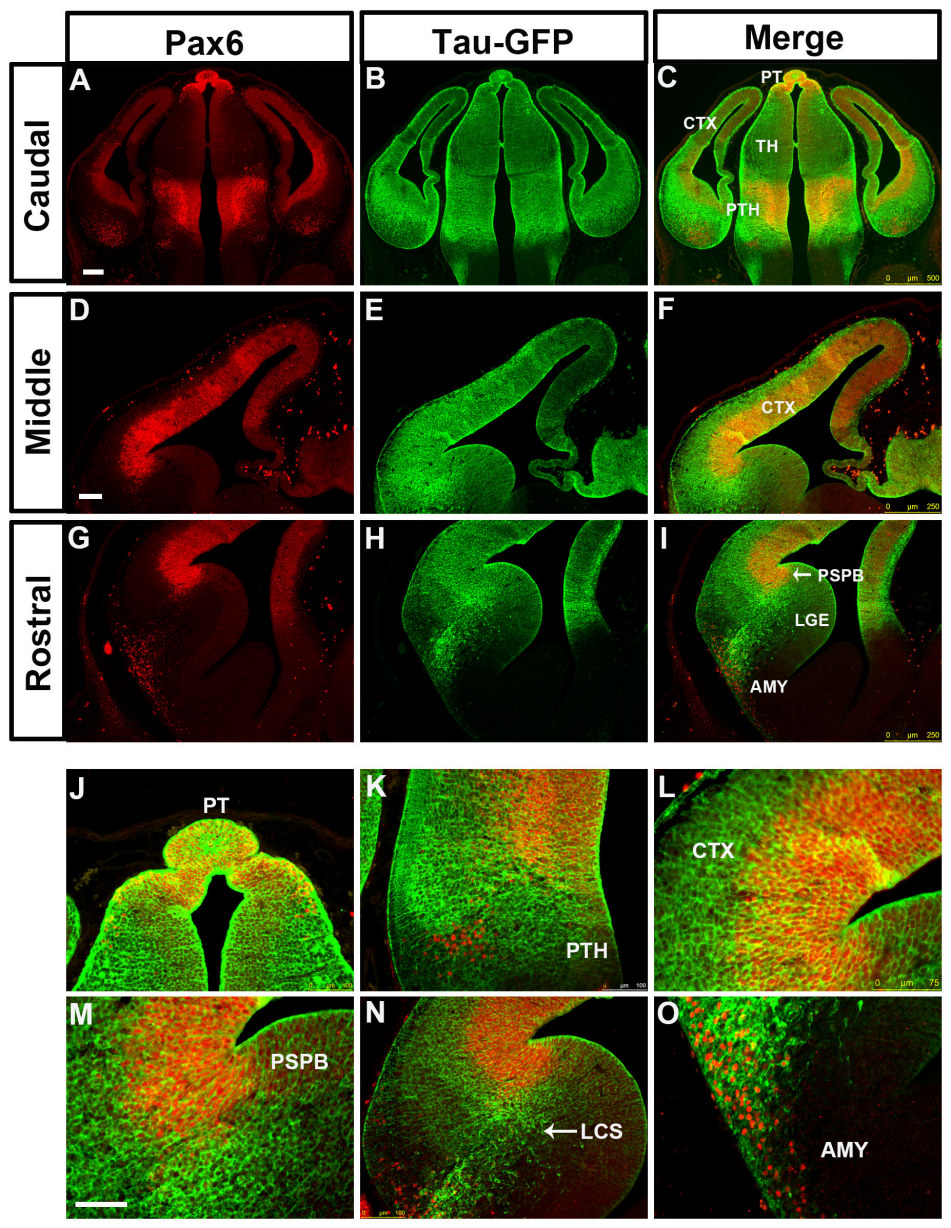
Double immunostaining for Pax6 and tau-GFP on coronal sections at caudal to rostral levels through the forebrain from E12.5 Y223 embryos. (A, D, G) Pax6 expression pattern is reflected by red fluorescence. (B, E, H) Tau-GFP is expressed in all Pax6 expressing regions. Tau-GFP heavy labeling of cellular processes can be seen in both the telencephalon and the diencephalon. (C, F, I) Direct comparison of tau-GFP and Pax6 expression revealed that tau-GFP expression replicates the known Pax6 pattern with some exceptions. (J) Pax6 and tau-GFP protein are co-localized in the pretectum (PT). (K) A group of cells only expressing Pax6 and not tau-GFP is intermingled with the tau-GFP labeled cellular processes in the prethalamus. (L) tau-GFP labeled radial glial processes extend into the mantle region of the cortex. (M) tau-GFP highly labeled cells are present on the subpallial side of the PSPB. (N) A subset of tau-GFP labeled migratory cells co-expresses Pax6. (O) Pax6 single expressing cells are present within the amygdaloid (AMY) region. Abbreviations as in Figure 2. n=10 Y223 embryos. Scale bars: A-C, 200µm; D-O, 100 µm.

To assess whether the integration site of the YAC transgene contributed to the discrepancy between Pax6 and tau-GFP expression in the DTy54 subpallium, we examined tau-GFP reporter expression in additional YAC transgenic lines. The transgenic line Y223 carries six copies of YAC Y374, which is slightly modified from YAC Y1123 by the insertion of two *loxP* sites [30](Figure 1B). Tau-GFP is expressed at particularly high levels in this transgenic line, most likely due to the higher copy number, but its pattern is otherwise identical to that in DTy54 mice (Figure 7), suggesting that neither the copy number, nor the genomic insertion site nor the presence of the *loxP* sites affects expression as compared to that of Y1123 (the YAC carried in line DTy54). The same discrepancy between the tau-GFP and the Pax6 expression in the subpallium was observed in this line as in DTy54 mice, indicating that the discrepancy is not due to the insertion site and is most likely due to the absence from the YAC of repressive elements required to silence Pax6 transcription in the subpallium. 

**Figure 8:**
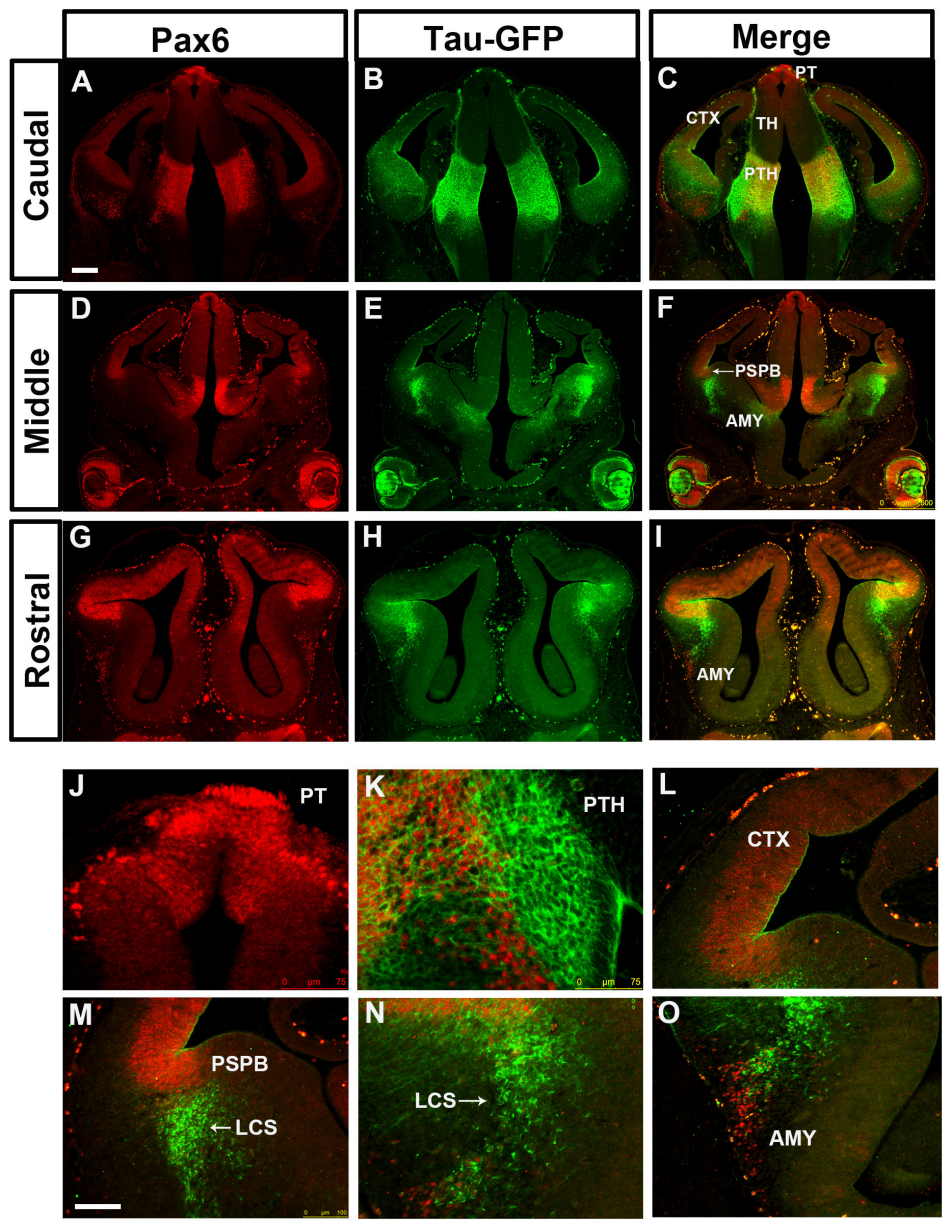
Double immunostaining for Pax6 and tau-GFP on coronal sections at caudal to rostral levels through the forebrain from E12.5 Y001 embryos. (A, D, G) Pax6 expression is reflected by red fluorescence. (B, E, H) In the diencephalon, tau-GFP is only expressed in the prethalamus (PTH). The tau-GFP expression in the cortex is dramatically reduced in comparison with the endogenous Pax6 expression. tau-GFP highly labeled cells are present in the ventral telencephalon, (C, F, I) Direct comparison of tau-GFP and Pax6 expression revealed that tau-GFP expression only exhibits a partial Pax6 pattern, with some notable discrepancies. (J) tau-GFP expression in the pretectum (PT) is lost. (K) A band of Pax6 only expressing cell is located adjacent to the tau-GFP labeled cellular processes in the prethalamus. (L) The expression of tau-GFP in the cortex (CTX) is very weak. (M) tau-GFP heavily labeled cells are present in the mantle region of the ventral telencephalon. (N) A subset of tau-GFP labeled migratory cells co-expresses Pax6. (O) Pax6 single expressing cells are present within the amygdaloid (AMY) region. Abbreviations as in Figure 2. n=8 Y001 embryos Scale bars: A-I, 200µm; J-O, 100 µm.

We then examined expression of tau-GFP in lines carrying truncated versions of Y374. Line Y001 carries a single copy of YAC 374 lacking approximately 20kb from its distal downstream end, lost during integration of the transgene [30,31] (Figure 1B). In the Y001 telencephalon, tau-GFP expression exhibits only a partial Pax6 expression pattern. In the cortex, the levels of tau-GFP expression appear much lower than those of Pax6 (Figure 8 A-I, L). In the Y001 diencephalon, tau-GFP expression is maintained in the prethalamus, but lost from the pretectum and the thalamus (Figure 8 A-F,J,K). These findings suggest that the missing distal 20kb contains *cis*-regulatory elements required for transgene expression in the cortex, thalamus and pretectum. In the Y001 subpallium, the same discrepancy between the tau-GFP and the Pax6 expression was observed as in DTy54 mice (Figure 8 D-I,M-O).

**Figure 9:**
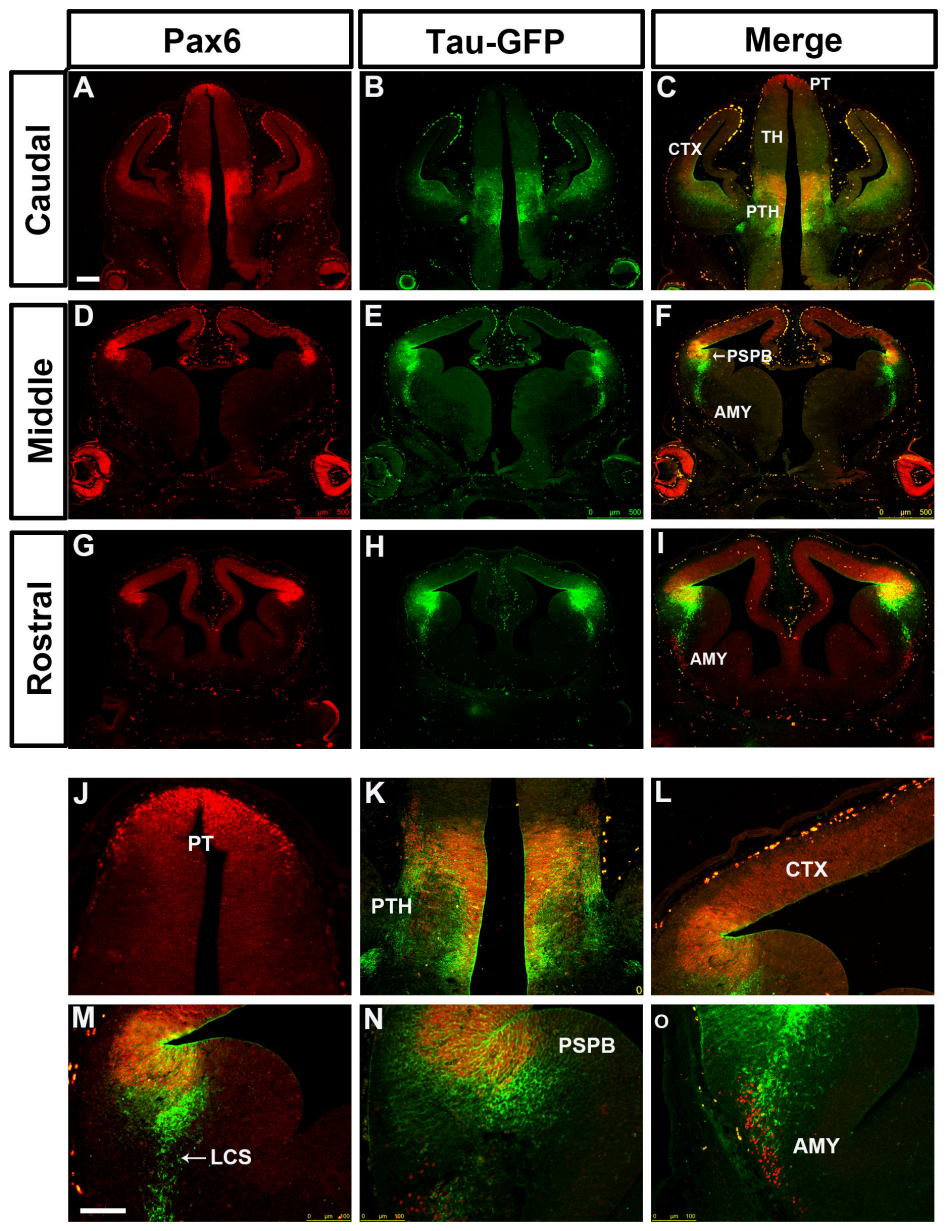
Double immunostaining for Pax6 and tau-GFP on coronal sections at caudal to rostral levels through the forebrain from E12.5 Y001ΔDRR embryos. (A, D, G) Pax6 expression is reflected by red fluorescence. (B, E, H) In the diencephalon, tau-GFP is only expressed in the prethalamus. The tau-GFP expression in the cortex is dramatically reduced in comparison with the endogenous Pax6 expression. tau-GFP highly labeled cells are present in the ventral telencephalon. (C, F, I) Direct comparison of tau-GFP and Pax6 expression revealed that tau-GFP expression only exhibits a partial Pax6 pattern, with some notable discrepancies. (J) Tau-GFP expression in the pretectum (PT) and the thalamus is lost. (K) tau-GFP expression in the prethalamus (PTH) is weak compared with the endogenous Pax6 expression. Tau-GFP labeling of cellular processes still can be clearly seen. (L) The expression of tau-GFP in the cortex (CTX) is very weak. (M) tau-GFP heavily labeled cells are present in the mantle region of the ventral telencephalon. (N) A subset of tau-GFP labeled migratory cells co-expresses Pax6. (O) Pax6 single expressing cells are present within the amygdaloid (AMY) region. Abbreviations as in Figure 2. n=8 Y001ΔDRR embryos. Scale bars: A-I, 200µm; J-O, 100 µm.

Transgenic mouse line Y001ΔDRR was generated previously from line Y001 by cre-mediated deletion of the 35 kb DRR, and therefore has the same genomic integration site [30] (Figure 1B). Expression of the transgene was very similar to that in Y001: levels of transgene expression were very low in the cortex, pretectum and thalamus, although contrary to previous conclusions [30] there was diencephalic expression in the prethalamus, as in Y001 (Figure 9 A-L). Some of the strongest tau-GFP expression was in the subpallium, where the same discrepancy between tau-GFP and Pax6 expression was observed as in DTy54 mice (Figure 9D-I,M-O).

In summary, these results indicate that elements distal to DRR are required for the expression of Pax6 in the cortex, thalamus and pretectum. Elements beyond even the full-length Y1123 are needed for correct regulation of Pax6 in the subpallium, involving repression in cells entering the LCS and expression in cells exiting it. 

### Tau-GFP reporter expression in the eyes of the YAC transgenic lines

**Figure 10:**
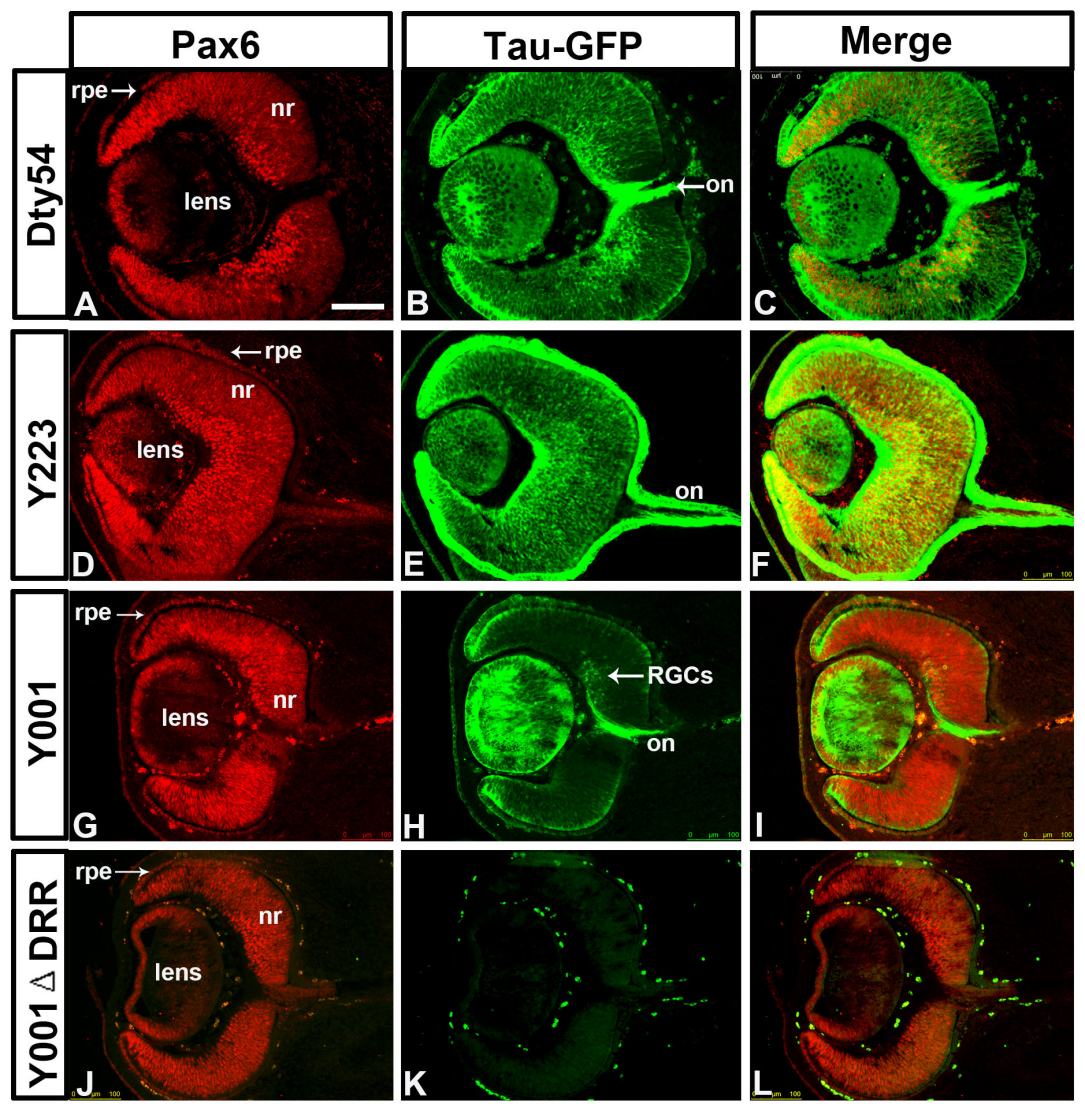
Double immunostaining for Pax6 and tau-GFP on coronal sections through the eye from E12.5 embryos of YAC transgenic mouse lines. (A, D, G, J) Pax6 is expressed in retinal pigmented epithelium (rpe), neural retina (nr) and lens. (B, C) In the DTy54 line, tau-GFP expression replicates the Pax6 pattern in all tissues of the eye. (E, F) In the Y223 line, tau-GFP expression matches Pax6 expression pattern in all eye tissues. (H, I) In the Y001 line, tau-GFP expression is restricted in the retina and is only present in some retinal ganglion cells (RGCs) that strongly express Pax6. (K, L) In the Y001ΔDRR line, Cre-mediated deletion of the DRR completely abolishes tau-GFP expression in the eye. (B, E, H) tau-GFP labeling of the axonal projections of RGCs can be seen in the optic nerve (on). n>8 YAC transgenic embryos per line. Scale bars: 100µm.

Pax6 is expressed dynamically during early development of the mouse eye, in both the surface ectoderm and the optic vesicle, which integrate to generate the eye [10,52-54]. At E12.5, Pax6 is widely expressed in the presumptive cornea, retinal pigmented epithelium, neural retina and lens (Figure 10 A,D,G,J). In both DTy54 and Y223 lines, tau-GFP and Pax6 expression coincided and retinal ganglion cell axons were labeled with tau-GFP in the optic nerve (Figure 10 B,C,E,F). In the Y001 line tau-GFP expression was maintained in most of these tissues (presumptive cornea, retinal pigmented epithelium, lens), but its level was reduced in many retinal cells (Figure 10 H,I). Only retinal ganglion cells that expressed the highest levels of Pax6 continued to express tau-GFP in this line, suggesting the existence of essential enhancer elements within the missing distal end of this YAC transgene that are required for full transgene expression in the retina. Deletion of the DRR in line Y001ΔDRR completely abolished transgene expression in all regions of the eye, consistent with previous observations and confirming the functional significance of the DRR in control of *Pax6* expression in the eye (Figure 10 K, L)[27,30]. 

## Discussion

The functional importance of the precise control of spatiotemporal levels of *Pax6* expression during embryonic development is well established. A number of evolutionarily conserved regulatory elements (CEs) in the *PAX6* locus have been described previously [25,27,30,35,55] and the finding that a YAC transgene containing a 420 kb region of the human *PAX6* locus rescued the mouse *small eye* (*Sey*) phenotype indicated that most of *PAX6*’s cis-acting regulatory elements are contained in this region [21]. Our new findings (i) indicate that at least one important element, which regulates *Pax6* expression in the LCS, lies outside this region and (ii) ascribe to a region within the 20kb downstream of the DRR a function in promoting high levels of cortical, pretectal and thalamic expression. 

### Analysis of tau-GFP reporter expression in YAC transgenic lines

We found that tau-GFP reporter expression in the forebrain and eye of two transgenic lines (DTy54 and Y223) carrying full-length reporter YAC transgenes derived from Y593 largely resembled that of Pax6, in agreement with previous studies [30,38]. However, close examination of tau-GFP and endogenous Pax6 expression at the cellular level highlighted some interesting differences. Some differences are most likely a consequence of the different intracellular locations of tau-GFP (cytoskeletal) and Pax6 (nuclear) proteins while others are likely due to differences between the half-lives of tau-GFP and Pax6 proteins. Other differences however, appear to be due to differences in the transcriptional regulation between the YAC transgenes and the endogenous *Pax6* locus. This is the most likely explanation for the presence of cells within the subpallial VZ that express very high levels of *tau-GFP* mRNA but only very low or no detectable *Pax6* mRNA. *Pax6* expression in this area is normally repressed by Gsh2 [41,56]. Therefore, one attractive possibility is that the Y1123 transgene may lack crucial repressor elements responsive to factors, possibly including Gsh2, that normally inhibit *Pax6* transcription in this region [56,57]. Differences between *Pax6* and *tau-GFP* mRNA expression were also found in the amygdaloid area and the prethalamus, where small groups of cells were seen to express *Pax6* mRNA, but not *tau-GFP* mRNA. All expression differences between *tau-GFP* and *PAX6* mRNA in the subpallial VZ, amygdaloid area and prethalamus were found in two separate transgenic strains containing almost identical regions of the *PAX6* locus, but integrated at different genomic positions, suggesting that the expression differences are due to the absence of cis-regulatory elements in the YAC DNA, rather than effects of the integration site on transgene expression (position effects). 

Examination of further transgenic lines, carrying truncated versions of Y374, confirmed the presence of essential regulatory elements at the distal end of the YAC. The YAC reporter transgene in line Y001, lacking the distal-most 20kb of YAC374, is expressed in the cortex (albeit at lower levels) and in the prethalamus, but not in the thalamus, consistent with previous reports [30,31], nor in the pretectum. The deleted 20kb region contains a highly conserved element, termed RB, which is able to drive expression of a reporter transgene in the diencephalon and the dorsal telencephalon [30]. It is interesting to note that although a previous study found that tau-GFP was widely expressed in the retina of Y001 embryos at E17.5 [30], here we found that many retinal ganglion cells in Y001 embryos did not express tau-GFP at E12.5, suggesting that unidentified regulatory elements within the very distal end of the YAC 374 are required for the temporal control of Pax6 expression in the retina.

In YAC transgenic line Y001ΔDRR, the 35 kb DRR present in line Y001 was deleted by cre-mediated recombination. Expression of the transgene was very similar to that in line Y001 in the telencephalon and diencephalon but was completely lost in the eye, indicating the functional importance of this genomic region in driving expression in the eye. A previous study indicated that the DRR is required for regulation of Pax6 expression in both the eye and diencephalon [30]. However, in the present study, detailed comparison between transgene and Pax6 expression provided novel evidence that unknown regulatory elements distal to DRR are more critical to drive expression in the diencephalon, since the loss of DRR in line Y001ΔDRR did not result in significant changes in transgene expression in the diencephalon. 

It is important to note that several eye and diencephalic regulatory elements have been characterized upstream of the DRR, including two lens enhancers located 5’ to *Pax6* P_*alpha*_ and P_4_ promoters [22,24,58], a diencephalic enhancer located between *Pax6* P_0_ and P_1_ promoters [22,26], a CE2 element within intron seven required for expression in both the eye and the diencephalon [25,28], and highly conserved elements E60 and E100 located downstream from *Pax6* exon 13 that drive expression in the diencephalon and retina [28]. The absence of tau-GFP expression in subregions of the eye and diencephalon in both Y001 and Y001ΔDRR lines suggests that unidentified regulatory elements within the distal-most 20kb of the YAC 374 are not redundant for the full Pax6 expression patterns in those regions of the brain. It is likely that full *Pax6* expression in the eye and diencephalon requires the actions of multiple *cis*-regulatory elements widely placed within the *Pax6* locus. In order to drive Pax6 expression, there might be a requirement for the activity of the regulatory elements within the very distal end of the full-length YAC transgene in coordination with other elements to drive overlapping expression patterns. At present, however, it is still unclear how exactly these *cis*-regulatory elements coordinate to control *Pax6* expression. Further study using directed mutations or deletions of specific enhancer elements in the intact YAC transgene or the endogenous *Pax6* locus will improve the understanding of their synergistic actions.

### Tau-GFP as a marker for the lateral cortical stream in Dty54 transgenic mice

The migratory cells of the lateral cortical stream (LCS) are derived from progenitors in the VZ of the PSPB and migrate ventrally towards the amygdaloid region where they contribute to the developing amygdala [47,50,59,60]. The LCS contains cells derived from both the pallium (Pax6+ and Tbr1+) and the subpallium (Dlx2+ and Gsh2+) [47,50,59]. Tau-GFP labels a set of Pax6-expressing cells which appear to be migrating towards the basolateral telencephalon of our *PAX6* YAC transgenic lines at both E12.5 and E15.5. Based on their location and expression of Pax6, Tbr1 and Gsh2, these almost certainly belong to the LCS. At E12.5, most of the tau-GFP labeled migrating cells were derived from the subpallial side of the PSPB. However, by E15.5, when migration of the LCS is most pronounced, tau-GFP labelled a broader population of migrating cells which comprised cells from both the pallium and subpallium, indicating that the cellular composition of the tau-GFP labeled LCS is dynamic. Our finding that tau-GFP labels multiple subsets of LCS cells during development demonstrates that these YAC transgenic strains may prove to be useful tools to visualize LCS migration defects in mutant mice.
